# Phenylpropane biosynthesis and alkaloid metabolism pathways involved in resistance of *Amorphophallus* spp. against soft rot disease

**DOI:** 10.3389/fpls.2024.1334996

**Published:** 2024-02-20

**Authors:** Penghua Gao, Ying Qi, Lifang Li, Shaowu Yang, Jianwei Guo, Jiani Liu, Huanyu Wei, Feiyan Huang, Lei Yu

**Affiliations:** College of Agronomy, Yunnan Urban Agricultural Engineering and Technological Research Center, Kunming University, Kunming, China

**Keywords:** konjac, phenylpropane biosynthesis, alkaloid metabolism, *Pectobacterium carotovorum* subsp. *carotovorum*, RNA-SeqTab

## Abstract

Soft rot of konjac (*Amorphophallus* spp.) is a devastating disease caused by the bacterium *Pectobacterium carotovorum* subsp. *carotovorum* (Pcc) with serious adverse effects on plantation development, corm quality and crop yield due to the current lack of effective control measures. The main objective of the present study was to elucidate the mechanisms underlying plant resistance to soft rot disease. A combination of transcriptomic and metabolomic analyses demonstrated significant enrichment of differentially expressed genes (DEG) and differentially accumulated metabolites (DAM) associated with plant hormones, phenylpropanoid biosynthesis and, in particular, alkaloid metabolism, in *Amorphophallus muelleri* following Pcc infection compared with *A. konjac*, these data implicate alkaloid metabolism as the dominant mechanism underlying disease resistance of *A. muelleri.* Quantitative real-time polymerase chain reaction analysis further revealed involvement of *PAL*, *CYP73A16*, *CCOAOMT1*, *RBOHD* and *CDPK20* genes in the response of konjac to Pcc. Analysis of the bacteriostatic activities of total alkaloid from *A. muelleri* validated the assumption that alkaloid metabolism positively regulates disease resistance of konjac. Our collective results provide a foundation for further research on the resistance mechanisms of konjac against soft rot disease.

## 1 Introduction

Konjac (*Amorphophallus* spp.), an economically important tuber taro species belonging to the family Araceae, is widely distributed among Asian countries including China, Japan and Burma. The corm of konjac is used as a thickening and gelation agent within the food processing, biotechnology, and pharmaceutical industries due to its high glucomannan content (Devaraj et al., 2019; Meng et al., 2021). Moreover, health protection effects of konjac through reduction of blood glucose and lipids levels and metabolic regulation have been reported (Wang et al., 2021). Due to its application prospects and economic value, planting of konjac has become the main pillar industry for farmers to lift themselves out of poverty in the Yunnan province of China. However, owing to expansion of the planting area, continuous cropping, and unreasonable preventive measures, outbreaks of konjac soft rot disease involving various symptoms, such as plants turning yellow and wilting, tissue decay, and emission of a foul odor, are a common occurrence (Wei et al., 2022). Soft rot disease can severely affect growth of *Amorphophallus* spp., resulting in a significant decrease in quality and yield (Charkowski, 2018). The pathogens of soft rot disease of *Amorphophallus* spp. have been identified as *Pectobacterium* spp. (Wei et al., 2019), *Dicyeya* spp (Wei and Wei, 2014), and *Erwinia* spp (Ban et al., 2009), of which *Pectobacterium carotovorum* subsp. *Carotovorum* (Pcc) is the prevalent disease-causing agent in the major planting areas of Kunming, Qujing, Chuxiong, and Yunnan provinces. Current strategies to control soft rot include application of pesticides and biocontrol (Cao et al., 2014). However, long-term use of chemical pesticides could not only increase the resistance of pathogenic bacteria but also cause serious damage to the ecological environment. Biocontrol is greatly influenced by both biotic and abiotic factors in the environment, resulting in unstable effects. One long-term and effective method of controlling plant diseases is to improve the plant’s own resistance. However, to our knowledge, few studies to date have focused on the mechanisms contributing to molecular resistance of *Amorphophallus* spp. against soft rot disease.

Under stress conditions, plants often enhance their resistance to disease-causing organisms by regulating the expression of specific genes and accumulation of metabolites. Metabolomics techniques can be effectively applied to assess changes in the metabolite profiles of plants during pathogen infection and identify a series of resistance-related metabolites. The data obtained not only inform further research into the defense mechanisms of plants but also facilitate the identification of functional genes involved in plant metabolism through comprehensive transcriptome analysis (Abdelrahman et al., 2018). Integration of transcriptomic and metabolomic analyses to predict the regulatory networks of biological traits and gene functions provides a valuable tool to evaluate the resistance response of plants to pathogenic infection. For example, salicylic acid metabolism was shown to be enriched in cabbage (*Brassica oleracea*) following infection with *Xanthomonas campestris* pv. *campestris* (*Xcc*) and the flavonoid pathway metabolites chlorogenic acid and caffeic acid effectively inhibited growth of *Xcc* (Sun et al., 2022). Flavonoid and steroidal saponin contents were increased while that of asparagusic acid glucose ester was decreased in resistant wild-type *Asparagus kiusianus* following infection with *Phomopsis asparagi* (Abdelrahman et al., 2020). Moreover, phenylpropanoids, flavonoids, alkaloids and terpenoid biosynthesis pathway resistance genes were shown to be strongly induced in association with resistance of potato (*Solanum tuberosum*) to late blight caused by *Phytophthora infestans* (Yogendra and Kushalappa, 2016). Therefore, comprehensive analysis of changes in gene expression patterns in the transcriptome and metabolite accumulation in the metabolome should facilitate elucidation of the regulatory mechanisms of plant responses to pathogen infection, which will be useful for breeding and quality improvement of disease-resistant crops.

Identification and utilization of the factors involved in resistance of *Amorphophallus* spp. may provide a basis for a viable control strategy for soft rot disease. During the cultivation process, we observed that compared to *Amorphophallus konjac*, *A. muelleri* showed stronger resistance to soft rot disease. Recently, our group conducted a preliminary study on the resistance of konjac to soft rot disease using non-parametric transcriptome analysis (Wei et al., 2022). However, since our team has successfully completed the whole genome sequencing of *A. konjac*, we re-applied the reference transcriptome to further explore the mechanisms underlying disease resistance of *Amorphophallus* spp. To this end, transcriptomics and metabolomics methodologies were employed to determine changes in gene expression profiles and metabolite accumulation between resistant and susceptible species. Moreover, correlations between transcriptomic and metabolomic data were examined with the expectation of developing a better understanding of the resistance mechanisms. Finally, real-time fluorescence quantitative polymerase chain reaction (qRT-PCR) and bacteriostasis test were conducted to validate the transcriptomic and metabolomic findings. This study provides valuable molecular information on the responses of *Amorphophallus* spp. to Pcc infection and the most significant biological pathways implicated in resistance against soft rot disease. Our collective findings should aid in greatly enhancing resistance breeding and biocontrol of plant growth and development and improving post-harvest storage of crops with higher resistance to *Amorphophallus* spp. soft rot disease.

## 2 Materials and methods

### 2.1 Plant growth and cultivation conditions

Three-month-old seedlings of *A. muelleri* and *A. konjac* were kindly provided by a Konjac germplasm resource nursery (Research Center for Engineering and Technology Urban Agricultural of Yunnan Province, Kunming, Yunnan Province, China; 24°97’E, 102°79’N). Each seedling was planted in a plastic pot (upper diameter of 20 cm, lower diameter of 12 cm, and depth of 14 cm) containing sterile nutrient soil and cultivated in an environmentally controlled greenhouse at Kunming University (Kunming, China) under a temperature of 27 ± 2°C, relative humidity of 80%, and a light/dark photoperiod of 16 h/8 h with a light intensity of 2000 Lux.

### 2.2 Pathogen inoculation and symptom analysis of soft rot disease

Bacteria (Pcc) causing soft rot disease of *A. muelleri* and *A. konjac* were stored at the Plant Pathology Laboratory of the Agriculture and Life Sciences College (Kunming University). Three-month-old seedlings of *A. muelleri* and *A. konjac* cultivated in greenhouses of Kunming University were used for inoculation. The surface of the inoculation site on a disease-free petiole of *A. muelleri* and *A. konjac* was cleaned with wet sterile cotton, and injected with Pcc bacterial suspension (50 µL; 10^8^ cfu/mL) using a disposable syringe. Inoculation sites injected with sterile Luria-Bertani (LB) medium served as the control group. One inoculation site was used for each plant (Wei et al., 2022). Sixty seedlings of each species of konjac were used for experiments, of which 15 uninfected seedlings were used as the control group. All inoculated seedlings were cultured in a greenhouse at Kunming University under the above cultivation conditions. Evaluation of soft rot disease in *A. muelleri* and *A. konjac* was conducted at 24 h and 48 h post-inoculation (hpi).

### 2.3 RNA-seq and data analysis

RNA-seq analyses of resistant *A. muelleri* (M) and susceptible *A. konjac* (K) species following Pcc infection at 0, 24 and 48 hpi were conducted, including control groups (inoculated with LB), which were designated MJ24, MJ48, KJ24, KJ48, MJ0 and KJ0 according to the time-point of study. Petiole samples were obtained at a 2.0 cm distance from the lesion spot (KJ24, KJ48, MJ24 and MJ48) or the inoculation site (KJ0 and MJ0). Three biological replicates were collected for each treatment. Each replicate contained mixed petiole samples of different seedlings from at least six sample sites. All samples were immediately frozen in liquid nitrogen and stored at −80°C until experimental use.

Total RNA was extracted using a TRIzol reagent kit (TransGen, Beijing, China) according to the manufacturer’s instructions, followed by evaluation with an Agilent 2100 Bioanalyzer (Agilent Technologies, Palo Alto, CA, USA). Agarose gel electrophoresis was employed to determine the quality of total RNA. Fragmentation of total RNA was followed by reverse transcription into cDNA using the NEBNext Ultra RNA Library Prep Kit for Illumina (NEB#7530; New England Biolabs, Ipswich, MA, USA). The purified product was subsequently ligated to Illumina sequencing adapters following the addition of A base. After purification of the ligation reaction, appropriately sized fragments were selected and sequenced using Illumina Novaseq6000 by Gene Denovo Biotechnology Co. (Guangzhou, China) to obtain a cDNA library. Firstly, fastp (version 0.18.0) (Chen et al., 2018) was utilized to remove data containing adapters, >10% unknown nucleotides (N) and >50% low quality (Q-value ≤ 20) bases from the raw data, resulting in clean data, followed by ribosome and reference genome alignment. Mapped reads of each test sample were reconstructed using StringTie v1.3.1 (Pertea et al., 2015, 2016) and fragment per kilobase of transcript per million mapped reads (FPKM) calculated using RSEM software (Li and Dewey, 2011). Principal component analysis (PCA) and Pearson correlation coefficient were applied to evaluate repeatability between samples. Differentially expressed genes (DEG) between the groups were analyzed with DESeq2 software (Love et al., 2014). Genes with false discovery rates (FDR) below 0.05 (FDR *P*-value < 0.05) and absolute fold change |log2FC| ≥ 2 were considered DEGs. To determine the potential functions of these genes and the metabolic pathways involved, Gene Ontology (GO) (Ashburner et al., 2000) and Kyoto Encyclopedia of Genes and Genomes (KEGG) (Kanehisa and Goto, 2000; Kanehisa, 2019; Kanehisa et al., 2023) were employed for enrichment analysis.

### 2.4 Metabolite extraction and data analysis

Metabolome analysis was conducted by Guangzhou Genedenovo Biotechnology Co., Ltd. (Guangzhou, China). The petiole was ground into powder in liquid nitrogen, and 80 mg of petiole powder extracted with 1 mL of methanol/acetonitrile/H2O (2:2:1, v/v/v) under low-temperature ultrasound for 60 mins, followed by incubation at −20°C for 10 min. Following centrifugation of the mixture for 20 mins (14000 g, 4°C), the supernatant obtained was vacuum-dried, dissolved in water and after filtration with a 0.22 μm microporous membrane prior to analysis on an Ultra High-Performance Liquid Chromatography System (UPLC, Vanquish UHPLC, ThermoFisher Technologies) using a 2.1 mm × 100 mm ACQUITY UPLC BEH 1.9 µm column (ThermoFisher Technologies). The mobile phase contained solvent A (25 mM ammonium acetate and 25 mM ammonium hydroxide in water) and solvent B (acetonitrile). The following gradient was used: 90% B for 1 min, linearly reduced to 65% in 11 min, further reduced to 40% in 0.1 min and maintained for 4 min, then increased to 85% in 0.1 min, with a 5 min re-equilibration period. The quality control (QC) sample was composed of equal volumes of all 18 test samples. QC samples were inserted into the sample queue for monitoring and evaluating the stability of the system and the reliability of experimental data. Primary and secondary spectra were collected with the AB Triple TOF 6600 mass spectrometry system (AB Sciex, USA). Qualitative analysis of metabolomic data was performed by searching the internal database using a self-compiled index (Guangzhou Genedenovo Biotechnology Co., Ltd, Guangzhou, China). Principal component analysis (PCA), partial least squares-discriminant analysis (PLS-DA), and orthogonal projection to latent structures-discriminant analysis (OPLS-DA) were performed on metabolomic data obtained using the corresponding R language package (Worley and Powers, 2013). Differentially accumulated metabolites (DAM) were screened based on t-test p values of < 0.05 and a variable importance in projection (VIP) ≥ 1. Finally, the functions of DAMs were annotated using the KEGG database (Kanehisa and Goto, 2000; Kanehisa, 2019; Kanehisa et al., 2023) to establish the pathways highly correlated with resistance of *Amorphophallus* spp. to Pcc infection.

### 2.5 Validation of gene expression

The sampled petioles of *A. muelleri* and *A. konjac* were the same as those used for RNA-seq analysis, specifically, MJ0, MJ24, MJ48, KJ0, KJ24 and KJ48 (**Supplementary Table S1**). Total RNA was extracted with a TRIzol reagent kit (TransGen, Beijing, China) and cDNA synthesized using TransScript^®^ One-Step RT-PCR SuperMix (TransGen). qRT-PCR was conducted using TransStart^®^ Top Green qPCR SuperMix (+Dye I) (TransGen). All protocols were performed according to the manufacturers’ instructions. The qRT-PCR assay was conducted in a total reaction volume of 20 μL under the following conditions: one cycle at 95°C for 30 s, 45 cycles at 95°C for 5 s, 60°C for 30 s and 72°C for 10 s. Three technical replicates were analyzed for each reaction. The actin gene of *A. konjac* was used as an internal reference and relative expression levels of the target genes were quantified using the 2^−(ΔΔCt)^ method (Livak and Schmittgen, 2001).

### 2.6 Bacteriostatic activities of alkaloids from *A. muelleri* on Pcc

The effects of alkaloids on propagates of Pcc were additionally examined. The Pcc propagation experiment was performed according to the methods of reported by Khan et al (Khan et al., 2022). and Ramkissoon et al (Ramkissoon et al., 2020), with minor modifications. Fresh konjac corms were dried at 50 °C to a constant weight, ground into powder, sieved through a 40 mesh sieve, and 1 kg of konjac powder was weighed and extracted in 95% alcohol for 24 hours (konjac powder: 95% alcohol = 1:20). Therefore, the alcohol extract is vacuum filtered using a vacuum rotary evaporator until it becomes viscous (temperature = 55°C). The concentrated viscous liquid was adjusted to pH 9.0 with 1mol/L NaOH, extracted five times with ethyl acetate, and concentrated again after merging the organic layers. The viscous liquid obtained from the final separation was total alkaloids. With spectrophotometer method, a total alkaloid content determination kit (Grace Biotechnology Co., Ltd., Suzhou, China) was used to determine the total alkaloid content of the obtained extract.

A stock solution of alkaloids (100 mg/mL) was prepared using methanol as the solvent and sterilized through a 0.22 μM microfilter. The final concentrations of alkaloids in LB were 0.1, 0.2, 0.4, 1 and 2 mg/mL. A control experiment was conducted by adding the corresponding volume of methanol into LB and the culture medium was used as the blank control. Bacterial suspensions (10 μL) at OD_600_ of 0.02 were added to 1 mL LB containing alkaloids and cultured at 27 ± 2°C and 150 g until the logarithmic growth phase of the blank control was reached. The absorbance value of bacterial suspensions was measured at a wavelength of 600 nm.

Under sterile conditions, the Pcc strain at the logarithmic stage was inoculated into LB liquid medium alone and that containing total alkaloid (1 mg/mL). Supernatant fractions were obtained via centrifugation (3400 g, 4°C, 10 min) after overnight incubation and absorbance values at 260 nm determined (Zhang, 2019.). Five replicates were analyzed for each treatment concentration of alkaloids and all experiments were repeated twice.

## 3 Results

### 3.1 Differences in soft rot disease between *A. muelleri* and *A. konjac*

**Figure 1:**
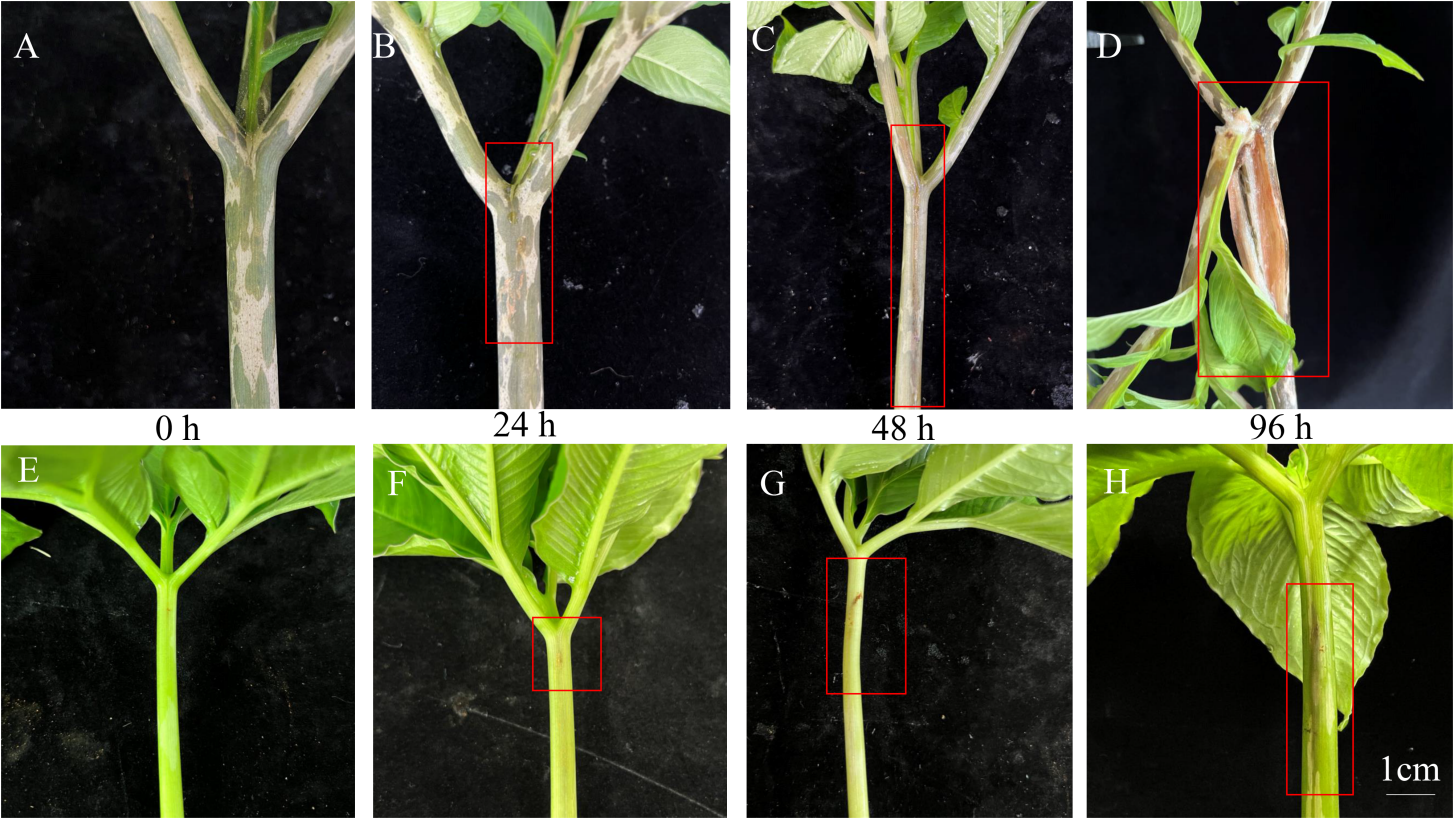
Symptoms of soft rot disease of *A. konjac* **(A–D)** and *A. muelleri* **(E–H)** at 0, 24, 48 and 96 h. The red box depicts the visual symptoms of soft rot disease.

Symptoms of soft rot disease in *A. muelleri* and *A. konjac* were used as indicators of plant resistance (**Figure 1**). After inoculation with Pcc for 0 h, the petioles of *A. konjac* and *A. muelleri* showed no symptoms (**Figures 1A, E**). With the development of disease after 24 hpi, *A. konjac* displayed water soaking with gummosis while no obvious symptoms were observed in *A. muelleri* (**Figures 1B, F**). Upon extension of the inoculation time, the diseased area of *A. konjac* expanded rapidly and *A. muelleri* presented slight soft rot symptoms (**Figures 1C, G**). After inoculation with Pcc for 96 h, the petioles of *A. konjac* showed considerable decay with progressive damage and death of the entire plant. At this time-point (**Figure 1D**), while the petioles of *A. muelleri* became hollow, growth was maintained (**Figure 1H**). The significant differences in disease symptoms between the species clearly indicate that *A. konjac* is more susceptible while *A. muelleri* is more resistant to Pcc infection.

### 3.2 Transcriptome analysis of *A. muelleri* and *A. konjac* petioles responsive to Pcc infection

**Figure 2:**
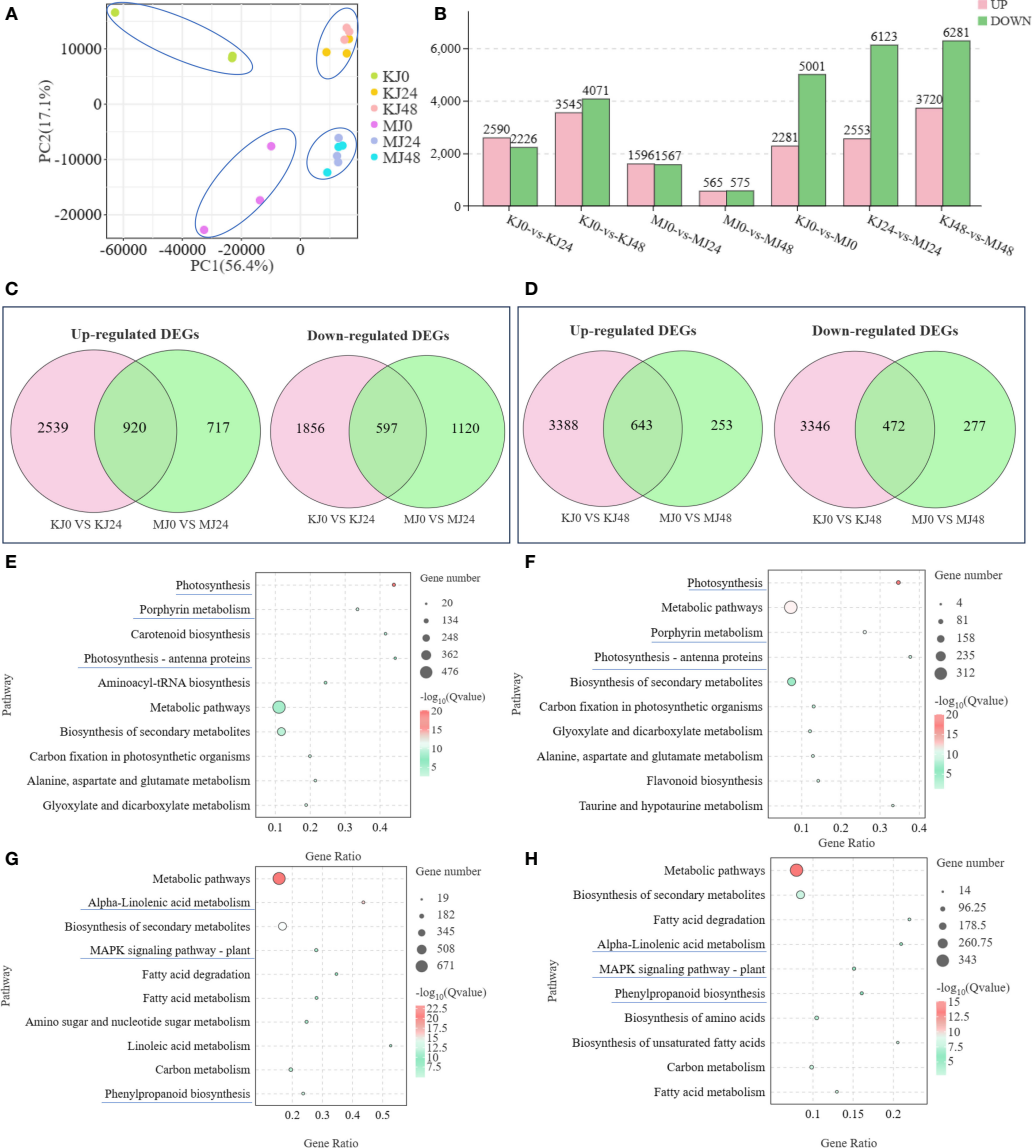
PCA analysis of expression of genes in KJ0, KJ24, KJ48, MJ0, MJ24 and MJ48 **(A)**. Overview of transcriptome analysis of A. muelleri and A. konjac responsive to Pcc infection. Bar graph of up- and down-regulated genes from pairwise comparisons **(B)**. Venn graph for up- and down-regulated DEGs from the pairwise comparisons of KJ0 vs KJ24 and MJ0 vs MJ24 **(C)**. Venn graph for up- and down-regulated DEGs from the pairwise comparisons of KJ0 vs KJ48 and MJ0 vs MJ48 **(D)**. KEGG enrichment bubble diagrams of down-regulated DEGs from the pairwise comparisons of KJ0 vs KJ24 and KJ0 vs KJ48 **(E)**. KEGG enrichment bubble diagrams of down-regulated DEGs from the pairwise comparisons of MJ0 vs MJ24 and MJ0 vs MJ48 **(F)**. KEGG enrichment bubble diagrams of up-regulated DEGs from the pairwise comparisons of KJ0 vs KJ24 and KJ0 vs KJ48 **(G)**. KEGG enrichment bubble diagrams of up-regulated DEGs from the pairwise comparisons of MJ0 vs MJ24 and MJ0 vs MJ48 **(H)**. We obtained the appropriate copyright permission to modify the KEGG image.

Three independent replicates of *A. muelleri* and *A. konjac* species responsive to Pcc infection at 24 hpi and 48 hpi were subjected to RNA-seq analyses, with a view to clarifying the potential mechanisms contributing to disease resistance. A total of 18 libraries (KJ0, KJ24, KJ48, MJ0, MJ24, and MJ48; three biological replicates per treatment) were combined into one pool, resulting in ~58.8 Gb clean data, a Q20 value above 97.58% and GC content between 50.95% and 52.52% (**Supplementary Table S2**). The data obtained could be successfully used for subsequent analyses. Principal component analysis (PCA) showed that the differences between control and infected plants were attributable to PC1 (56.4%) and those between *A. muelleri* and *A. konjac* to PC2 (17.1%) (**Figure 2A**). The correlation between the three biological replicates for each treatment was >0.75, indicating significant similarities in gene expression patterns (**Supplementary Figure S1**). Seven comparative DEG analyses (KJ0 *vs* KJ24, KJ0 *vs* KJ48, MJ0 *vs* MJ24, MJ0 *vs* MJ48, KJ0 *vs* MJ0, KJ24 *vs* MJ24 and KJ48 *vs* MJ48) revealed 4816, 7616, 3163, 1140, 7282, 8676 and 10001 DEGs, respectively. Among these, 2590, 3545, 1596, 565, 2281, 2553 and 3720 genes were up-regulated while 2226, 4071, 1567, 575, 5001, 6123 and 6281 genes were down-regulated in response to infection, respectively, clearly demonstrating that infection with Pcc induces significant changes in plant gene expression patterns (**Figure 2B**).

Venn diagram analysis showed that 920 common genes were up-regulated in KJ0 vs KJ24 and MJ0 vs MJ24, whereas 597 common genes were down-regulated (**Figure 2C**); 643 common genes were up-regulated in KJ0 vs KJ48 and MJ0 vs MJ48, whereas 472 common genes were down-regulated (**Figure 2D**). Additionally, 717 and 253 up-regulated DEGs were found in MJ24 and MJ48, respectively, whereas 2539 and 3346 up-regulated DEGs were found in KJ24 and KJ48, respectively (**Figures 2C, D**). These up-regulated DEGs, unique to MJ24 and MJ48, may be involved in resistance against bacterial infection.

To explore the key pathways activated by Pcc infection, KEGG annotations of up-regulated and down-regulated DEGs of *A. konjac* and *A. muelleri* infected with Pcc. The results showed that in both *A. konjac* and *A. muelleri*, the down-regulated genes were mainly enriched in pathways of “photosynthesis,” “photosynthesis-antenna protein” and “porphyrin metabolism” (**Figures 2E, F**) while up-regulated genes were the most highly represented in “biosynthesis of secondary metabolites,” “alpha-linolenic acid metabolism,” “MAPK signaling pathway-plant,” and “phenylpropanoid biosynthesis” pathways (**Figures 2G, H**). These findings support the conclusion that Pcc infection activates *A. konjac* and *A. muelleri* resistance responses and induces the expression of genes associated with metabolic pathways implicated in disease resistance.

**Figure 3:**
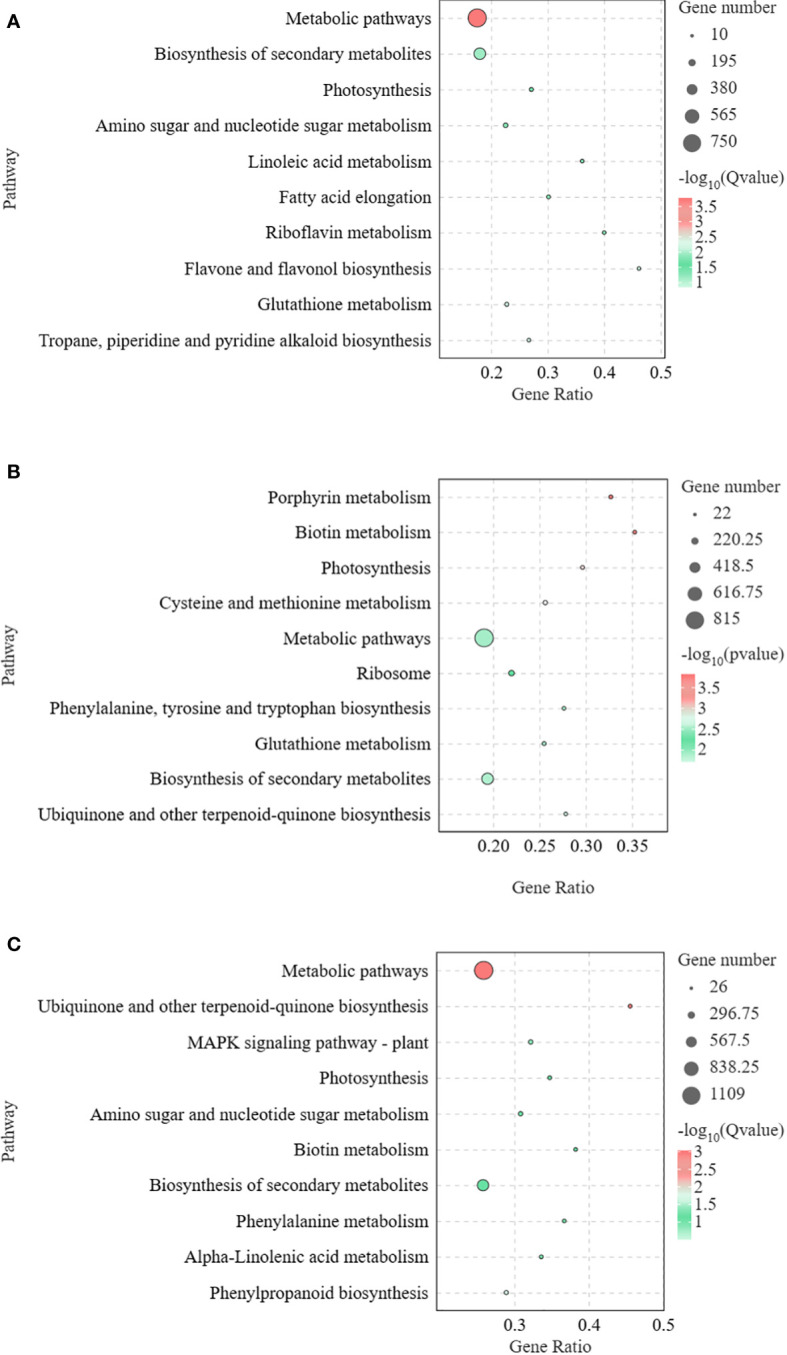
KEGG enrichment bubble diagrams of DEGs between KJ0 and MJ0 **(A)**, KJ24 and MJ24 **(B)**, KJ48 and MJ48 **(C)**. We obtained the appropriate copyright permission to modify the KEGG image.

To clarify the functions of DEGs at different time points of Pcc infection in *A. muelleri* and *A. konjac*, KEGG annotations of these genes were analyzed between groups (KJ0 vs MJ0, KJ24 vs MJ24, and KJ48 vs MJ48). DEGs between KJ0 and MJ0 were mainly enriched in “metabolic pathways,” “biosynthesis of secondary metabolites,” “flavone and flavonol biosynthesis,” “tropane, piperidine and pyridine alkaloid biosynthesis” and “glutathione metabolism” (**Figure 3A**). DEGs between KJ24 and MJ24 were mainly enriched in “ubiquinone and other terpenoid-quinone biosynthesis,” “glutathione metabolism,” “biosynthesis of secondary metabolites” and “ubiquinone and other terpenoid-quinone biosynthesis” (**Figure 3B**). DEGs between KJ48 and MJ48 were mainly enriched in “metabolic pathways,” “ubiquinone and other terpenoid-quinone biosynthesis,” “isoquinoline alkaloid biosynthesis,” “MAPK signaling pathway-plant,” “phenylpropanoid biosynthesis” and “amino sugar and nucleotide sugar metabolism” (**Figure 3C**). The results support the potential involvement of “MAPK signaling pathway-plant,” “phenylpropanoid biosynthesis,” “glutathione metabolism” and alkaloid metabolic pathways in the response of konjac to Pcc.

**Figure 4:**
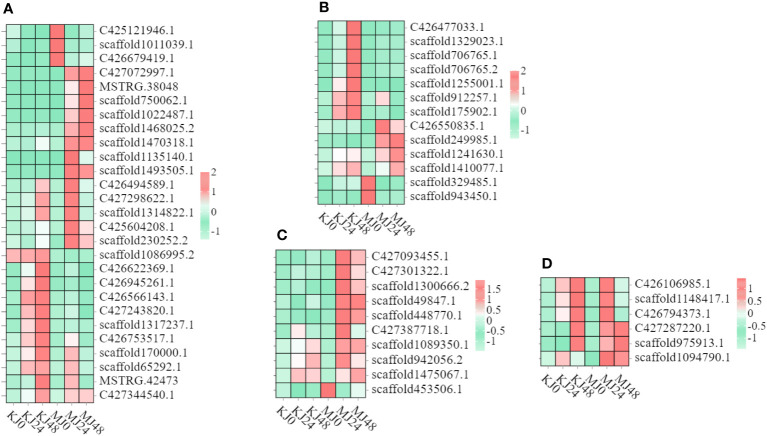
Heatmap of genes in *A*. *muelleri* and *A*. *konjac* in response to Pcc infection. The bar represents the scale of the expression relative abundance for each gene in the different treatments, as indicated by pink/green rectangles. Genes in pink show upregulation, and those in green show downregulation. **(A)** Phenylpropanoid biosynthesis pathway genes. **(B)** Isoquinoline alkaloid biosynthesis pathway genes. **(C)** Ca^2+^ signal pathway genes. **(D)** respiratory burst oxidase genes.

Heatmaps of DEGs subclusters were developed to better understand the key DEGs associated with the resistance of konjac to Pcc (**Supplementary Table S3**). The resulting heatmaps showed DEGs involved in plant-pathogen interactions. Based on their functional annotation, these genes included 27 phenylpropanoid biosynthesis pathway genes (**Figure 4A**), 13 isoquinoline alkaloid biosynthesis pathway genes (**Figure 4B**), four calcium-binding protein genes, six calcium-dependent protein kinase genes (**Figure 4C**), and six respiratory burst oxidase genes (**Figure 4D**).

### 3.3 Metabolome analysis of *A. muelleri* and *A. konjac* petioles responsive to Pcc infection

**Figure 5:**
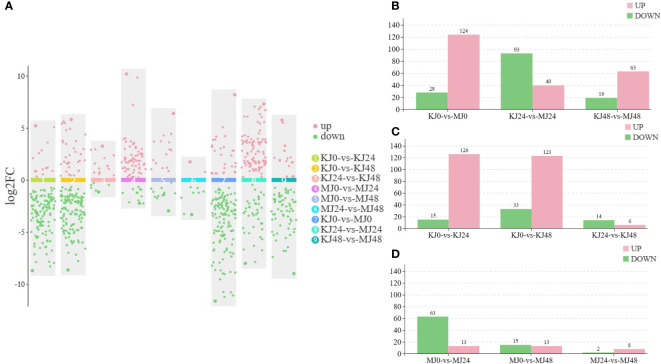
Overview of metabolomic analysis of *A*. *muelleri* and *A*. *konjac* in response to Pcc infection. **(A)** Scatter plot of multiple comparison groups. **(B)** Histogram of the number of DAMs between *A*. *muelleri* and *A*. *konjac* after Pcc infection at 0, 24 and 48 hpi. **(C, D)** Histogram of the number of DAMs in *A*. *konjac* **(C)** and *A*. *muelleri* **(D)** at distinct post-infection time-points.

A non-targeted metabolome analysis was performed on Pcc-infected *A. muelleri* and *A. konjac* petiole samples at 0, 24 and 48 hpi, designated M0, MJ24, MJ48 and KJ0, KJ24 and KJ48, respectively. A total of 1658 metabolites (1047 in the positive ion mode (POS) and 611 in the negative ion mode (NEG) were identified from all samples. The orthogonal Projections to Latent Structures Discriminant Analysis (OPLS-DA) method was employed for subsequent model tests and DAM screening (**Supplementary Figure S2**). Based on VIP ≥1 and t-test P <0.05, DAMs were identified (**Figure 5A**). After Pcc infection, the number of DAMs in susceptible *A. konjac* was higher than that in resistant *A. muelleri*. Further comparative analysis of each group revealed a total of 152 DAMs between KJ0 and MJ0, among which 28 metabolites were up-regulated and 124 were down-regulated in MJ0 relative to KJ0. Comparison of KJ24 and MJ24 revealed a total of 133 DAMs, among which 93 metabolites were up-regulated and 40 were down-regulated in MJ24. Comparison of KJ48 and MJ48 led to the identification of 82 DAMs, among which 19 metabolites were up-regulated and 63 were down-regulated in MJ48 (**Figure 5B**). The collective data clearly indicate significant differences between the metabolomes of *A. muelleri* and *A. konjac* petioles following Pcc infection.

Overall, 141 DAMs were detected between KJ0 and KJ24, with 15 up-regulated and 126 down-regulated metabolites at 24 hpi. Comparison of KJ0 and KJ48 revealed a total of 156 DAMs, among which 33 metabolites were up-regulated and 123 were down-regulated at 48 hpi (**Figure 5C**). We detected 76 DAMs between MJ0 and MJ24, among which 63 metabolites were up-regulated and 13 were down-regulated at 24 hpi. A total of 28 DAMs were identified in comparative analysis of MJ0 and MJ48, with 15 up-regulated and 13 down-regulated metabolites at 48 hpi (**Figure 5D**). The DAMs in *A. muelleri* and *A. konjac* petioles responsive to Pcc infection were mainly classified into 10 categories, specifically, organic acids and derivatives, alkaloids and derivatives, phenylpropanoids and polyketides, organoheterocyclic compounds, lipids and lipid-like molecules, organic oxygen compounds, benzenoids, nucleotides and analogs, lignans, neolignans and related compounds, and others (**Supplementary Table S4**).

**Figure 6:**
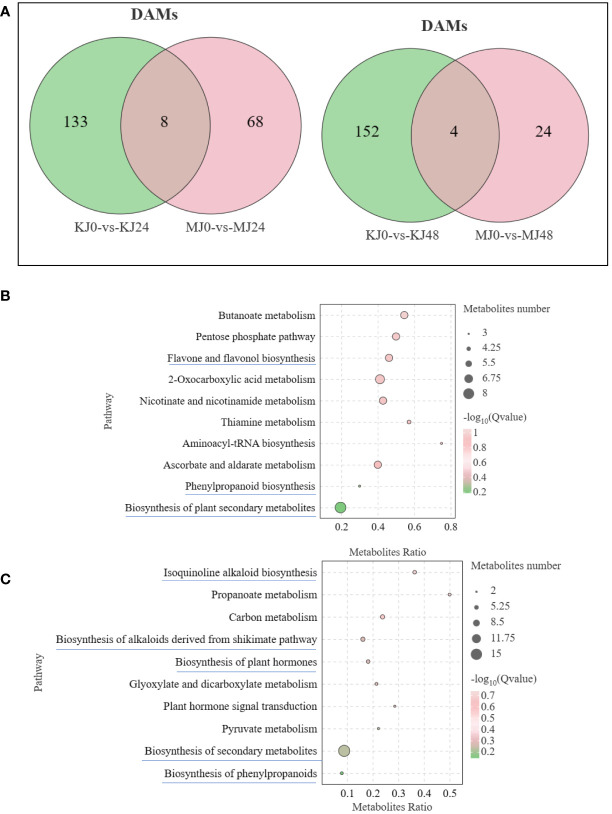
**(A)** Venn graph for DAMs from the pairwise comparisons of KJ0 vs KJ24 and MJ0 vs MJ24 and KJ0 vs KJ48 and MJ0 vs MJ48. **(B)** KEGG enrichment bubble diagrams of DAMs from the pairwise comparisons of KJ0 vs KJ24 and KJ0 vs KJ48. **(C)** KEGG enrichment bubble diagrams of DAMs from the pairwise comparisons of MJ0 vs MJ24 and MJ0 vs MJ48. We obtained the appropriate copyright permission to modify the KEGG image.

Venn diagram analysis showed that 8 common DAMs in KJ0 vs KJ24 and MJ0 vs MJ24 and 4 common DAMs in KJ0 vs KJ48 and MJ0 vs MJ48. Additionally, 68 and 24 DAMs were uniquely found in MJ24 and MJ48, respectively; and 133 and 152 DAMs were uniquely found in KJ24 and KJ48, respectively (**Figure 6A**). These DAMs may be involved in resistance against bacterial infection.

To understand the main pathways activated by Pcc infection, KEGG annotations of these unique DAMs of *A. muelleri* and *A. konjac* were analyzed. In *A. konjac*, The DAMs were mainly involved in “Flavone and flavonol biosynthesis,” “biosynthesis of phenylpropanoids” and “biosynthesis of secondary metabolites,” (**Figure 6B**); In *A. muelleri*, three cluster modes were significantly enriched and their DAM contents markedly increased compared with MJ0. These DAMs were mainly involved in “isoquinoline alkaloid biosynthesis,” “biosynthesis of plant secondary metabolites,” “Biosynthesis of alkaloids derived from shikimate pathway,” “Biosynthesis of plant hormones,” and “phenylpropanoid biosynthesis” pathways (**Figure 6C**). Interestingly, DAMs of the “biosynthesis of plant secondary metabolites” pathway were mainly involved in synthesis of alkaloids and plant hormones. Our results suggest that “phenylpropanoid biosynthesis,” “biosynthesis of plant hormones” and “biosynthesis of plant secondary metabolites” pathways are potentially involved in the defense mechanisms of *A. muelleri* and *A. konjac*.

DAMs between KJ0 and MJ0 were mainly enriched in “tropane, piperidine and pyridine alkaloid biosynthesis,” “nicotinate and nicotinamide metabolism” and “indole alkaloid biosynthesis” pathways (**Supplementary Figure S3**). DAMs between KJ24 and MJ24 were mainly enriched in “glycerophospholipid metabolism,” “monoterpenoid biosynthesis” and “indole alkaloid biosynthesis” and DAMs between KJ48 and MJ48 were mainly enriched in “tryptophan metabolism,” “diterpenoid biosynthesis,” “biosynthesis of alkaloids derived from shikimate pathway” and “biosynthesis of plant secondary metabolites” (**Supplementary Figure S3**). The collective results indicate that alkaloid metabolites may participate in *A. muelleri* resistance to Pcc infection.

### 3.4 Involvement of the phenylpropanoid biosynthesis pathway in resistance of *A. muelleri* and *A. konjac* against soft rot disease

**Figure 7:**
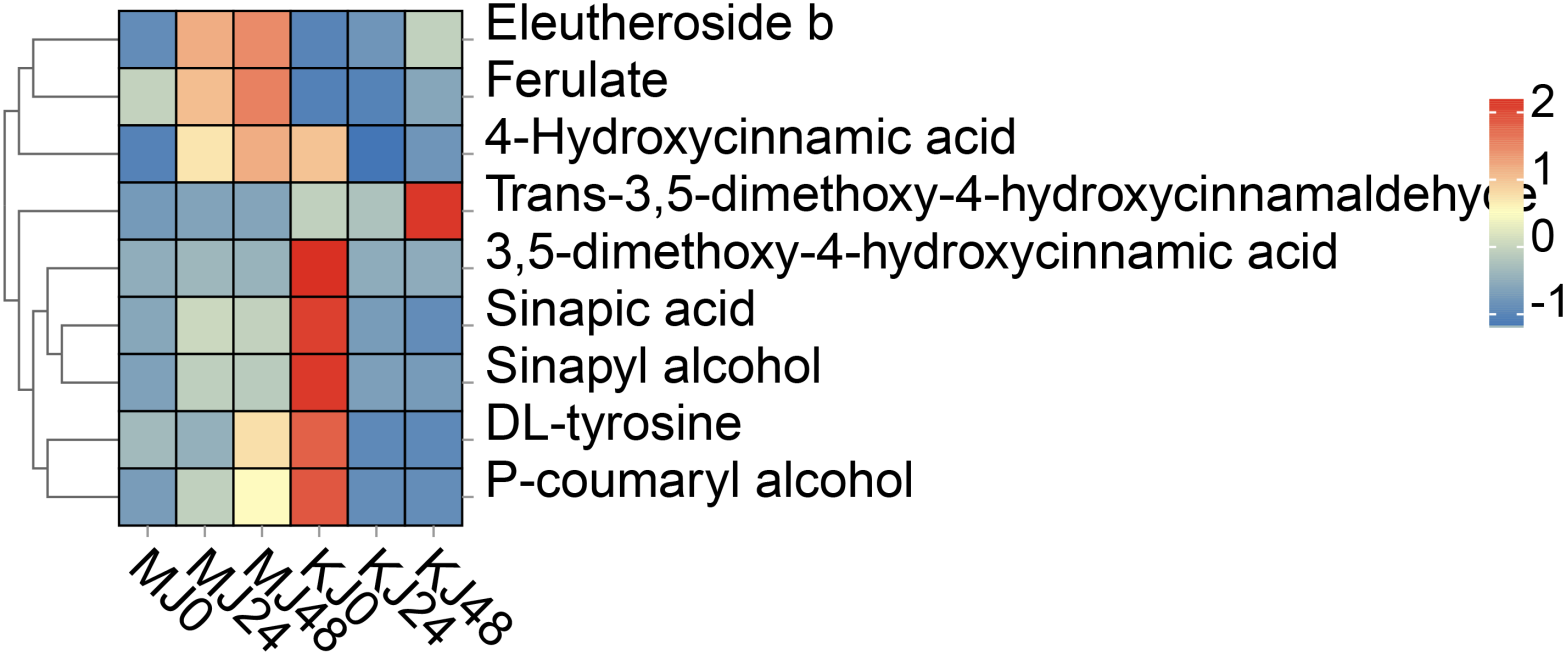
DAMs in phenylpropanoid biosynthesis in Pcc-infected *A. muelleri* and *A. konjac* at 0, 24 and 48 hpi.

Phenylpropanoid metabolism is one of the most important mechanisms in plants, with multiple branching pathways producing flavonoids, coumarins, lignans, lignin, and other metabolites that participate in growth and development and response to various biotic and abiotic stresses stimuli (Dong and Lin, 2021). In this study, a total of nine phenylpropanoid metabolites were identified, including cinnamic acids and derivatives (4), cinnamyl alcohols (1), phenols (1) and other phenylpropane metabolites (3). Compared with MJ0, the contents of ferulic acid, 4-hydroxycinnamic acid and eleutheroside b were significantly increased and p-coumaryl alcohol, sinapic acid and sinapyl alcohol levels relatively increased in MJ24 and MJ48. No marked differences in the other metabolites were observed among the groups. However, these metabolites exhibited different patterns in *A. konjac*. Compared with KJ0, the contents of ferulic acid, trans-3,5-dimethoxy-4-hydroxycinnamaldehyde and eleutheroside b in KJ24 and KJ48 were significantly increased while those of other metabolites were significantly decreased (**Figure 7**). The comprehensive results of these analyses indicate that Pcc infection triggers the phenylpropanoid metabolism process of *A. muelleri* and *A. konjac*, leading to changes in the contents of secondary metabolites of this pathway.

### 3.5 qRT-PCR-based validation of candidate DEGs

**Figure 8:**
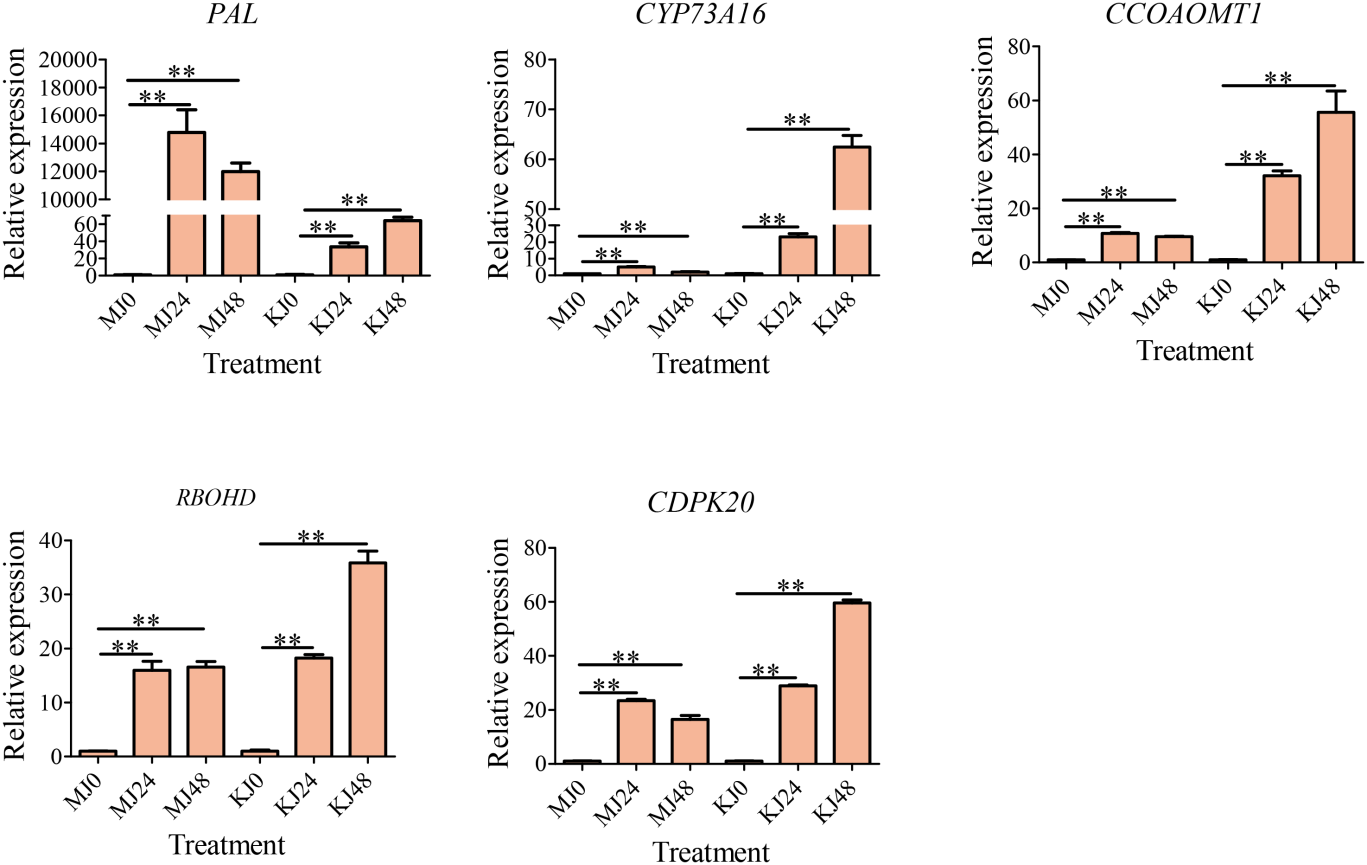
qRT-PCR-based verification of the expression profiles of five unigenes. ** indicate significant differences at the P<0.01 level.

The phenylpropanoid metabolism pathway produces secondary metabolites, such as lignans, lignin, and flavonoids, through a series of enzymatic reactions that participate in plant growth, development, and stress response. Phenylalanine amine lyase, trans-cinnamate 4-monooxygenase, and caffeoyl-CoA O-methyltransferase are key enzymes in the phenylpropanoid metabolic pathway in plants and are involved in the biosynthesis of secondary metabolites, such as lignin and flavonoids. *CYP73A16s* and *CCOAOMT1s* are involved in the synthesis of phenylalanine ammonia-lyase, trans-cinnamate 4-monooxygenase, and caffeoyl-CoA O-methyltransferase. Therefore, to determine whether candidate DEGs were involved in the responses of *A. muelleri* and *A. Konjac* to Pcc, the expression of three phenylpropane biosynthesis pathway genes (*PAL*, *CYP73A16* and *CCOAOMT1*) was screened via qRT-PCR (**Figure 8**). Furthermore, an oxidative stress-related gene (*RBOHD*) and a calcium ion channel-related gene (*CDPK20*) were screened by qRT-PCR (**Figure 8**). All five genes were significantly induced in *A. muelleri* and *A. Konjac* in response to infection with Pcc, supporting their utility as candidates for further evaluation of the disease resistance mechanism of konjac.

### 3.6 Involvement of total alkaloid of *A. muelleri* on Pcc growth

**Figure 9:**
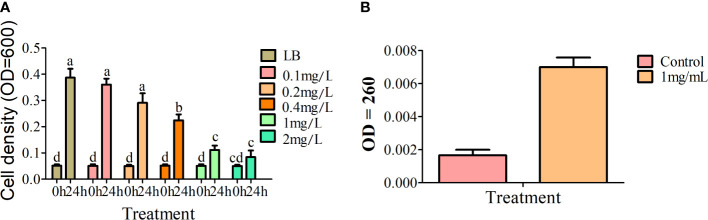
Effects of extracts of *A. muelleri* and *A. konjac* on growth of Pcc. **(A)** Effects of total alkaloid from *A. muelleri* on growth of Pcc. **(B)** Effects of 1mg/mL total alkaloid from *A. muelleri* on membrane permeability of Pcc. Different letters indicate significant differences at the P<0.01 level.

Alkaloids are important bioactive substances in plants that participate in defense against a variety of biotic and abiotic stress conditions. Transcriptome and metabolome analyses suggest that alkaloids of *A. muelleri* play an important role in inhibiting growth of Pcc. To establish the effect of total alkaloid from *A. muelleri* on Pcc growth, total alkaloid was isolated from healthy petioles for antibacterial experiments, with an extract yield of ~2.76 mg/g. Compared with the control group, alkaloids from *A. muelleri* inhibited the growth of Pcc in a dose-dependent manner. At alkaloid concentrations of >1 mg/mL, the cell density of Pcc was only one-third that of the control Pcc group (**Figure 9A**). The cell membrane maintains cellular integrity and is the basis of energy metabolism. Following damage, the cell membrane releases nucleic acids, which absorb light at a wavelength of 260 nm. Growth of Pcc was markedly inhibited and the OD_260_ value was significantly higher than that of the control group after incubation for 24 h with 1 mg/mL total alkaloid (**Figure 9B**), indicating that total alkaloids of *A. muelleri* cause damage to the cell membrane of Pcc, resulting in nucleic acid leakage. Our findings strongly support the theory that alkaloids in *A. muelleri* serve as a contributory factor to its resistance to soft rot disease.

### 3.7 Co-joint analysis

A co-joint KEGG enrichment analysis showed 42, 23, 48, and 63 co-mapping pathways across the pairwise comparisons of KJ0 vs KJ24, KJ0 vs KJ48, MJ0 vs MJ24, and MJ0 vs MJ48, respectively (**Supplementary Table S5**). Pearson correlation coefficients were used to explore the correlations between DEGs and DAMs. The correlations between the top 250 DEGs and their related DAMs were selected and represented as a heat map (**Supplementary Table S6**; **Supplementary Figure S4**).

## 4 Discussion

Long-term and effective strategies to control plant diseases can be achieved by improving the resistance of plants themselves. Soft rot disease is widely prevalent throughout the whole planting area of *Amorphophallus* spp. Our conclusions are consistent with earlier reports that *A. muelleri* is resistant while *A. konjac* is susceptible to soft rot disease (Wei et al., 2022). Screening and identification of species resistant to *Amorphophallus* spp. through artificial inoculation should provide a foundation for further research on the defense mechanisms of plants against soft rot disease.

**Figure 10:**
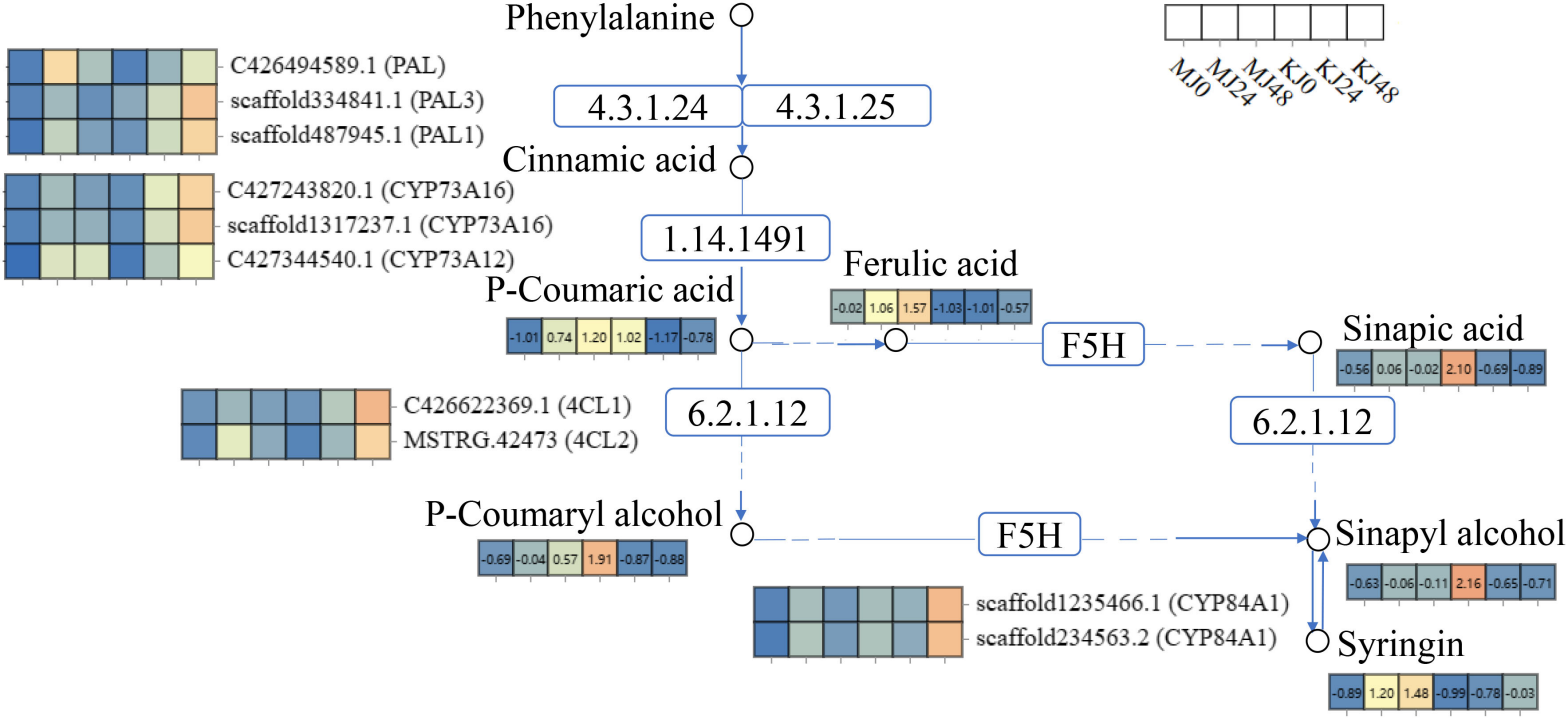
Expression profiles of genes and metabolites involved in the phenylpropanoid biosynthesis pathway of *A. muelleri* and *A. konjac* petioles responsive to Pcc infection. The rectangle patterns with non-numbered rectangles represent the genes and the numbered rectangle represent the metabolites. Differential expression of each annotated gene and metabolite is presented as a heatmap on the corresponding place of the gene or metabolite, with the scale ranging from low (blue) to high (orange). The white rectangles represent the sample name.

During plant-bacteria interactions, complex systems in plants are activated to participate in the defense response to pathogens. The plant mitogen-activated protein kinase (MAPK) cascade reaction (Lin et al., 2022), hormones (Verma et al., 2016), and secondary metabolites, such as alkaloids, flavonoids, phenolic acids (Zaynab et al., 2018), are collectively involved in mediating plant defense against pathogen infection. The MAPK cascade activates downstream transcription factors through a series of protein phosphorylation reactions, triggering various defense reactions in plants (Lu et al., 2020; Zhou et al., 2020, 2022; Zhao et al., 2023). In addition, the MAPK cascade reaction interacts with a host of signaling pathways, such as plant hormones (Wang et al., 2023), reactive oxygen species (Zhang et al., 2023), calmodulins, and calcium-dependent protein kinases (Wurzinger et al., 2011), to regulate the same defense process, forming a complex defense network. Plant hormones have been shown to induce defense genes, regulate the synthesis of secondary metabolites, and participate in the resistance response to pathogens (Gupta et al., 2020; Huang et al., 2022). In this study, DEGs were enriched in the pathways of “alpha-linolenic acid metabolism,” “glutathione metabolism,” “phenylpropanoid biosynthesis,” and “MAPK signaling pathway - plant” in *A. muelleri* and *A. konjac*. In response to Pcc infection, downstream defense genes of pathogen pattern-triggered immunity (PTI) were induced, including *MAPK*, respiratory burst oxidase (*RBOH*), calmodulin (*CML*), calcium-dependent protein kinase (*CDPK*), *WRKY* transcription factors, abscisic acid receptor (*PYL*, *PP2C*) and ethylene receptor (*ETR*, *EIN*). Recently, our group showed that methyl jasmonate administered as a spray could enhance the resistance of *A. konjac* to Pcc (Gao et al., 2022). Here, “alpha-linolenic acid metabolism” pathway genes were extensively activated that participated in the synthesis of jasmonic acid (JA). Glutathione relies on both its antioxidant capacity and modulation of plant hormones to regulate plant defense against pathogens (Zhu et al., 2021). Our results showed induction of “glutathione metabolism” pathway genes in response to oxidative stress induced by Pcc. The “phenylpropanoid biosynthesis” pathway is closely related to plant resistance to disease and its phenylalanine ammonia-lyase (PAL) activity serves as a critical physiological indicator for assessing resistance. Following Pcc infection, PAL and lignin synthesis-related (4-coumarate-CoA ligase (*4CL*), 5-O-(4-coumaroyl)-D-quinate 3’-monooxygenase (*CYP98A*)) and defense (peroxidase (*POD*), beta-glucosidase (*BGLU*)) genes of *A. muelleri* and *A. konjac* were induced (**Figure 10**; **Supplementary Table S7**). Taken together, the results suggest that konjac forms a complex defense network system through activating plant hormones, regulating signal transduction processes, and inducing disease resistance and antioxidant genes that collectively participate in resistance to Pcc infection.

Following pathogenic infection, plants ultimately synthesize antitoxins that function in resistance through changes in their metabolites (DuFall and Solomon, 2011). Previous study showed that alkaloids are important antibacterial components of plants (Zielińska et al., 2019; Li et al., 2020). And the intermediate metabolites of phenylpropanoid biosynthesis pathways are related to plant defense against pathogens (Ahuja et al., 2012; Costa et al., 2016; Ranjan et al., 2019).Under Pcc stress, up-regulated DAMs were mainly enriched in disease-resistant metabolic pathways, including “biosynthesis of plant hormones,” “biosynthesis of plant secondary metabolites” and “biosynthesis of phenylpropanoids,” in both *A. muelleri* and *A. konjac.* Interestingly, we observed an increase in the alkaloid metabolite content in *A. muelleri.* Our results are in keeping with earlier findings implicating phenylpropanoid and indole alkaloids in the resistance response of rice (*Oryza sativa*) to *Xanthomonas oryzae* pv. *oryzicola* (Tang et al., 2022), *Pueraria lobata* to *Synchytrium puerariae Miy* (Huang et al., 2022), and *Musa acuminata* to Fusarium wilt (*Fusarium oxysporum f.* sp. *cubense*) (Sun et al., 2019).

In response to Pcc infection, the contents of intermediate metabolites in biosynthesis of phenylpropanoids of *A. muelleri* and *A. konjac* exhibited different trends. We propose that these differences in alkaloid and phenylpropanoid metabolites potentially contribute to the stronger resistance of *A. muelleri* to Pcc relative to *A. konjac.*

Alkaloids are natural products widely present in animals and plants that have a range of physiological properties, such as insecticidal, antifungal, anti-tumor, and cytotoxic activities (Thuy et al., 2012; Sledz et al., 2015). Earlier, Kubo et al. (2022) isolated a jerveratrum-type steroidal alkaloid from *Veratrum californicum* with inhibitory effects on *Botrytis cinerea* and *Puccinia recondita.* Similarly, the group of Zielińska et al. (2019) reported antibacterial activities of seven main alkaloids in an extract of *Chelidonium majus* (Zielińska et al., 2019). In the present study, compared with *A. konjac*, DAMs in Pcc-infected *A. muelleri* were mainly enriched in two alkaloid metabolism pathways, specifically, “biosynthesis of alkaloids derived from shikimate pathway” and “isoquinoline alkaloid biosynthesis.” Harmine and mescaline levels were significantly increased in both *A. muelleri* and *A. konjac*, with relatively higher contents in *A. muelleri*. Moreover, Pcc infection promoted the expression of genes in the alkaloid metabolism pathway in both *A. muelleri* and *A. konjac*, including polyphenol oxidase (*PPO*), (S)-stylopine/(S)-canadine/(S)-nandinine synthase (*CYP719A13*), and aspartate-prephenate aminotransferase (*AAT*) genes. *In vitro* analysis of the antibacterial activity of total alkaloids from *A. muelleri* against Pcc showed that LB containing 0.4 mg/mL total alkaloids could effectively inhibit bacterial growth. Our results are consistent with previous reports showing that alkaloids function in plant resistance to pathogens.

## 5 Conclusion

RNA-seq and metabolomic analyses were effectively employed to elucidate the defense mechanisms of *Amorphophallus* spp. against Pcc infection. Under Pcc-induced stress, the following disease resistance-related pathways were activated: “alpha-linolenic acid metabolism,” “isoquinoline alkaloid biosynthesis,” “MAPK signaling pathway-plant” and “phenylpropanoid biosynthesis.” Moreover, *PAL*, *CYP73A16*, *CCOAOMT1*, *RBOHD* and *CDPK20* genes were implicated in the response of *Amorphophallus* spp. to Pcc. Consistently, metabolomic results revealed the involvement of both phenylpropanoid biosynthesis and alkaloid metabolism pathways in response to infection. Interestingly, following infection with Pcc, the numbers of DEGs and DAMs in resistant *A. muelleri* were lower than those in *A. konjac*. These differential alterations in the phenylpropanoid and alkaloid metabolite contents between *A. muelleri* and *A. konjac* after Pcc infection could explain the stronger resistance of *A. muelleri*. Total alkaloid of *A. muelleri* was identified as a component that could effectively inhibit Pcc growth.

## Data availability statement

The datasets presented in this study can be found in online repositories. The names of the repository/repositories and accession number(s) can be found below: BioProject, PRJNA1017648.

## Author contributions

PG: Writing – original draft, Writing – review & editing. YQ: Funding acquisition, Software, Writing – review & editing. LL: Investigation, Software, Writing – review & editing. SY: Methodology, Writing – review & editing. JG: Methodology, Writing – review & editing. JL: Resources, Writing – review & editing. FH: Conceptualization, Formal analysis, Writing – original draft, Writing – review & editing. LY: Funding acquisition, Resources, Supervision, Writing – original draft, Writing – review & editing. HW: Methodology, Writing – review & editing.

## Supplementary material

Supplementary Figure 1 Correlation heat map of expression of genes in KJ0, KJ24, KJ48, MJ0, MJ24 and MJ48.

Supplementary Figure 2 OPLS-DA analysis of DAMs for KJ0, KJ24, KJ48, MJ0, MJ24 and MJ48.

Supplementary Figure 3 KEGG enrichment bubble diagrams of DAMs between KJ0 and MJ0 **(A)**, KJ24 and MJ24 **(B)**, and KJ48 and MJ48 **(C)**. We obtained the appropriate copyright permission to modify the KEGG image.

Supplementary Figure 4 Heatmap of the top 250 DEGs and relevant metabolites.
