# Corrigendum: Phenylpropane biosynthesis and alkaloid metabolism pathways involved in resistance of Amorphophallus spp. against soft rot disease

**DOI:** 10.3389/fpls.2024.1404082

**Published:** 2024-04-18

**Authors:** Penghua Gao, Ying Qi, Lifang Li, Shaowu Yang, Jianwei Guo, Jiani Liu, Huanyu Wei, Feiyan Huang, Lei Yu

**Affiliations:** College of Agronomy, Yunnan Urban Agricultural Engineering and Technological Research Center, Kunming University, Kunming, China

**Keywords:** konjac, phenylpropane biosynthesis, alkaloid metabolism, Pectobacterium carotovorum subsp. carotovorum, RNA-SeqTab

In the published article, there was an error in the legend for **Figure 2** as published. We mistakenly typed the comparison group in **Figure 2G**. The sentence originally stated: “KEGG enrichment bubble diagrams of up-regulated DEGs from the pairwise comparisons of MJ0 vs MJ24 and MJ0 vs MJ48”. The corrected legend appears below.

**Figure 2:**
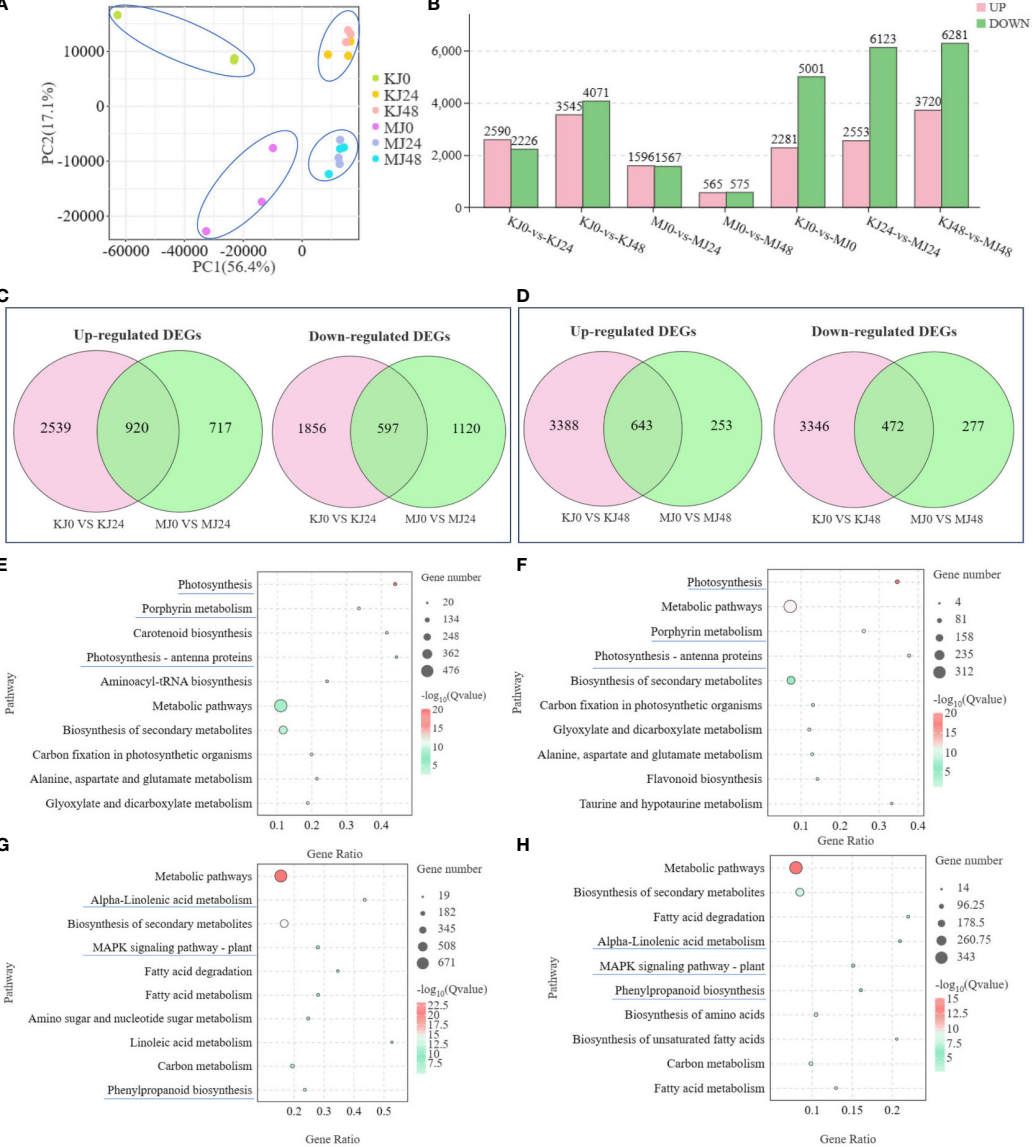
PCA analysis of expression of genes in KJ0, KJ24, KJ48, MJ0, MJ24 and MJ48 **(A)**. Overview of transcriptome analysis of *A. muelleri* and *A. konjac* responsive to Pcc infection. Bar graph of up- and down-regulated genes from pairwise comparisons **(B)**. Venn graph for up- and down-regulated DEGs from the pairwise comparisons of KJ0 vs KJ24 and MJ0 vs MJ24 **(C)**. Venn graph for up- and down-regulated DEGs from the pairwise comparisons of KJ0 vs KJ48 and MJ0 vs MJ48 **(D)**. KEGG enrichment bubble diagrams of down-regulated DEGs from the pairwise comparisons of KJ0 vs KJ24 and KJ0 vs KJ48 **(E)**. KEGG enrichment bubble diagrams of down-regulated DEGs from the pairwise comparisons of MJ0 vs MJ24 and MJ0 vs MJ48 **(F)**. KEGG enrichment bubble diagrams of up-regulated DEGs from the pairwise comparisons of KJ0 vs KJ24 and KJ0 vs KJ48 **(G)**. KEGG enrichment bubble diagrams of up-regulated DEGs from the pairwise comparisons of MJ0 vs MJ24 and MJ0 vs MJ48 **(H)**. We obtained the appropriate copyright permission to modify the KEGG image.

The authors apologize for this error and state that this does not change the scientific conclusions of the article in any way. The original article has been updated.

