# Supplementary figures and images for: Phenylpropane biosynthesis and alkaloid metabolism pathways involved in resistance of Amorphophallus spp. against soft rot disease
Source: Front Plant Sci. 2024 Feb 20;15:1334996. doi: 10.3389/fpls.2024.1334996 (PMC10912172; doi:10.3389/fpls.2024.1334996)

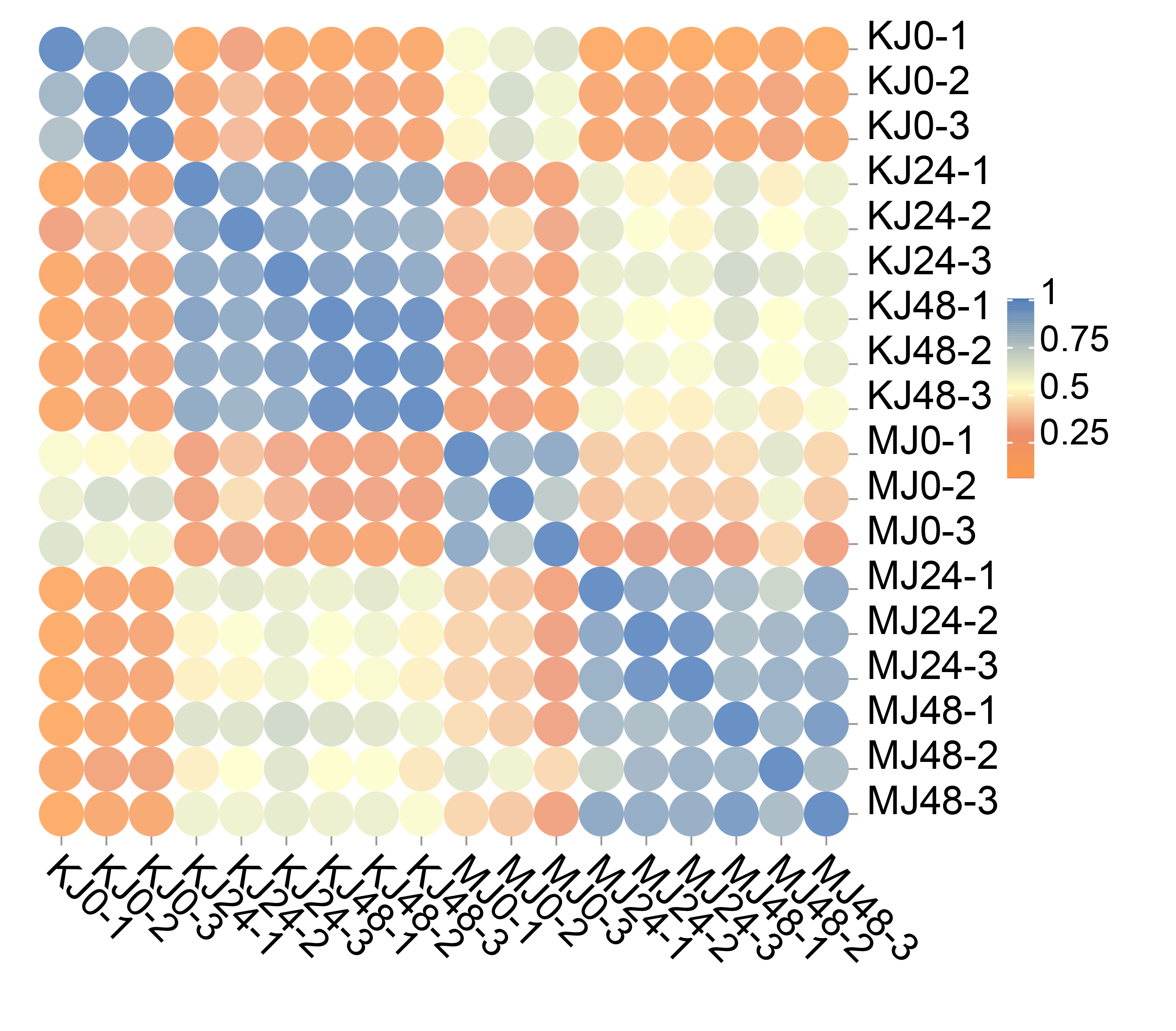

Supplement: Supplementary Figure 1 — Correlation heat map of expression of genes in KJ0, KJ24, KJ48, MJ0, MJ24 and MJ48. [file Image_1.tif]

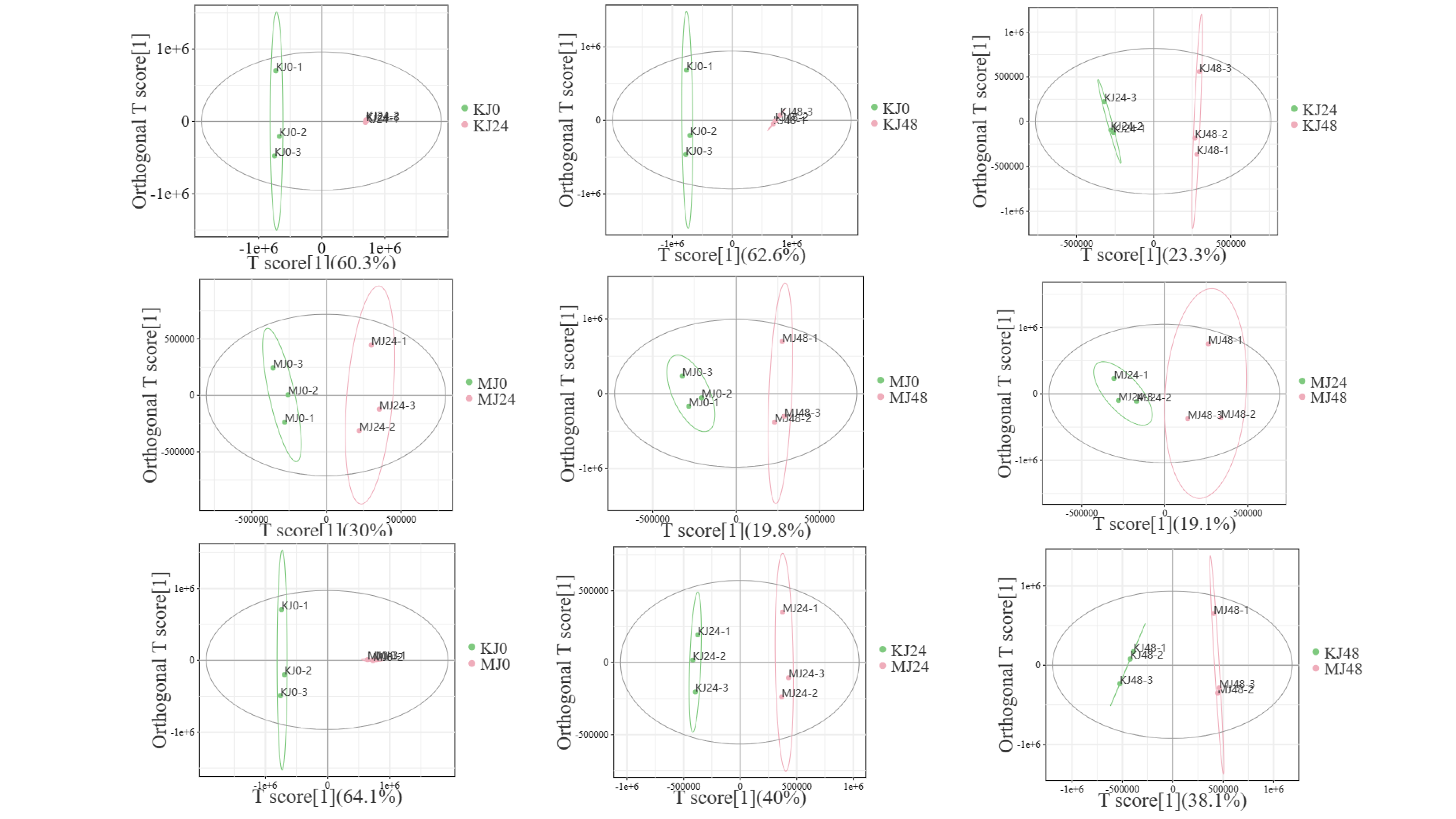

Supplement: Supplementary Figure 2 — OPLS-DA analysis of DAMs for KJ0, KJ24, KJ48, MJ0, MJ24 and MJ48. [file Image_2.tif]

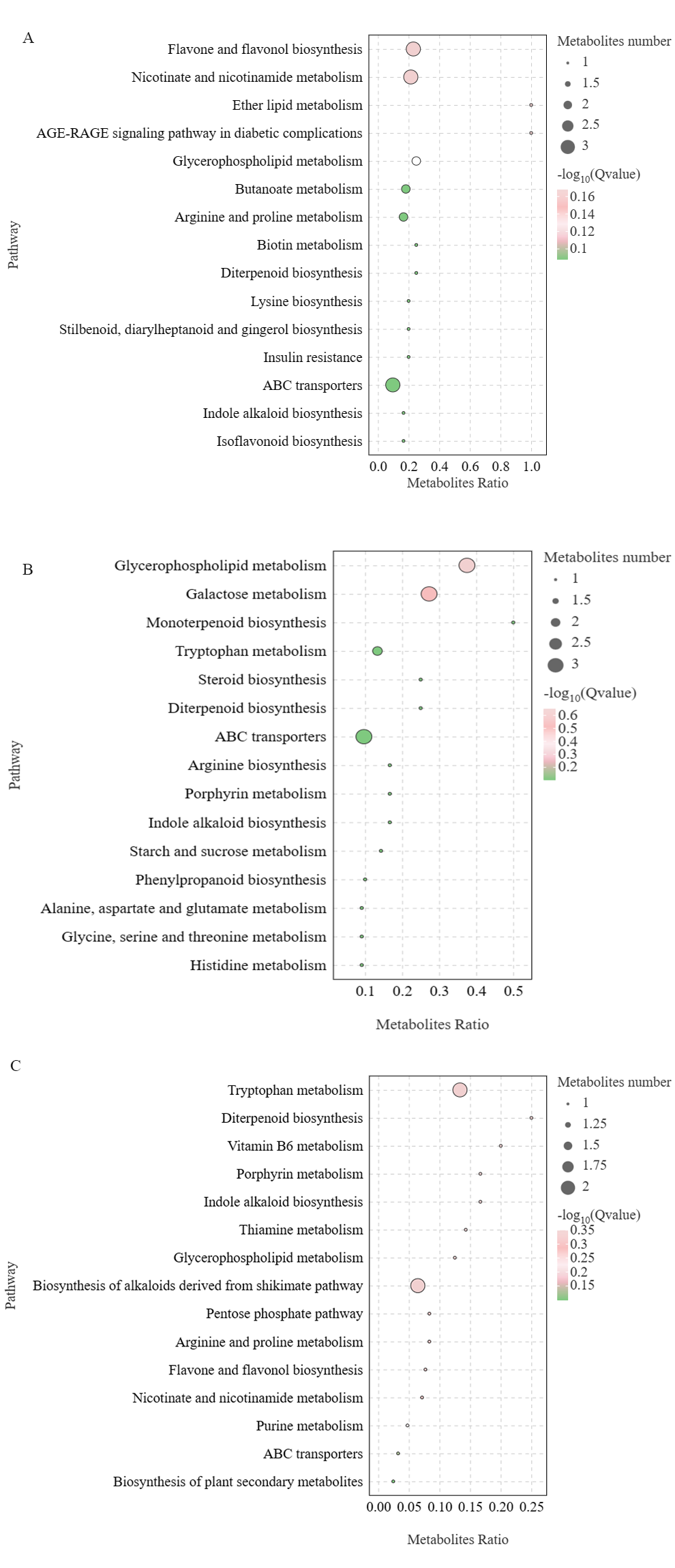

Supplement: Supplementary Figure 3 — KEGG enrichment bubble diagrams of DAMs between KJ0 and MJ0 (A), KJ24 and MJ24 (B), and KJ48 and MJ48 (C). We obtained the appropriate copyright permission to modify the KEGG image. [file Image_3.tif]

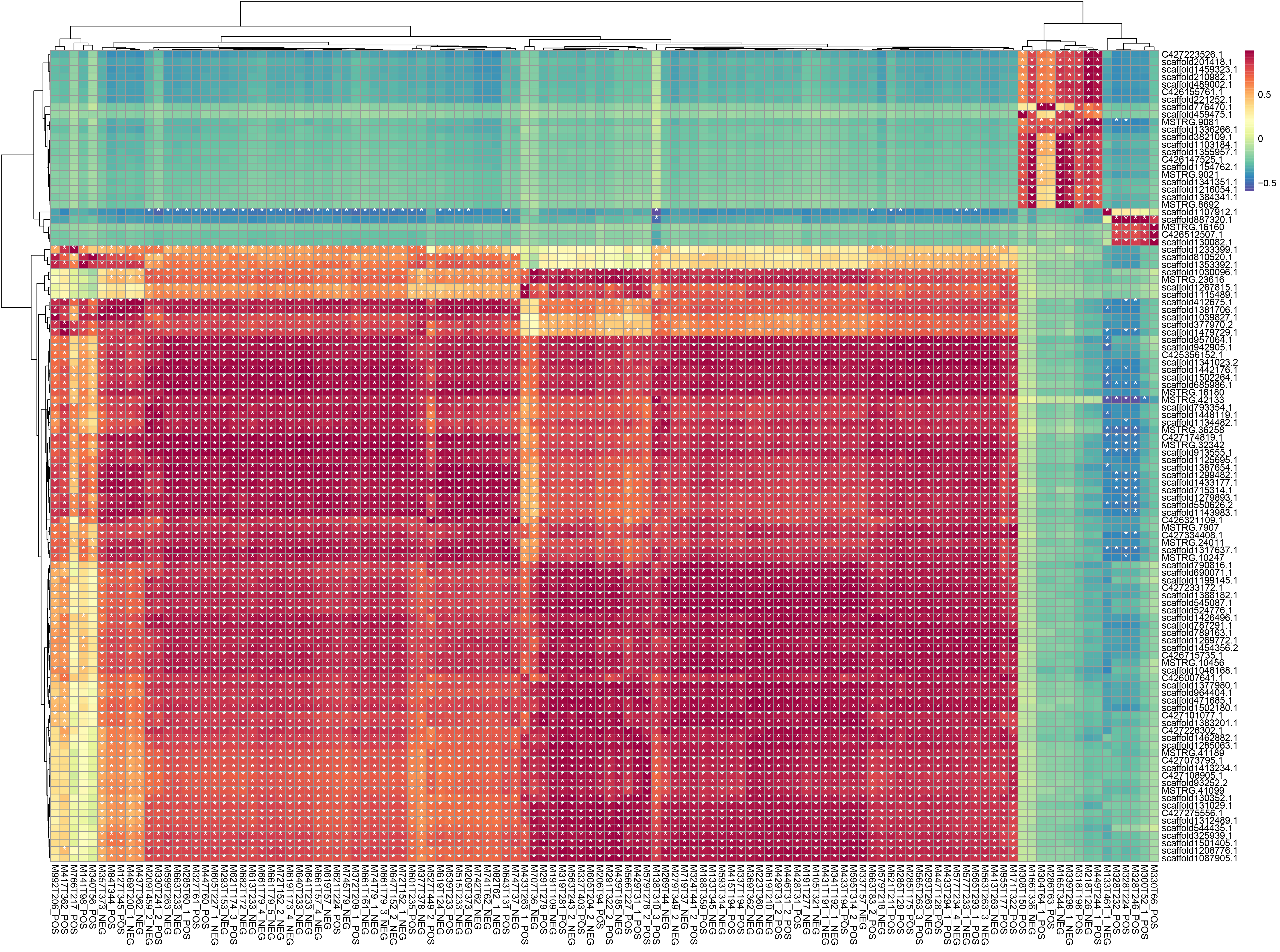

Supplement: Supplementary Figure 4 — Heatmap of the top 250 DEGs and relevant metabolites. [file Image_4.tif]
